# Underutilization of biomarker testing and DMT treatment among patients with MCI due to suspected Alzheimer’s disease

**DOI:** 10.1002/alz.089633

**Published:** 2025-01-09

**Authors:** Margaret Stabb, Sarah Soucy

**Affiliations:** ^1^ RxY, New York, NY USA

## Abstract

**Background:**

Disease‐modifying therapies (DMTs) such as lecanemab and aducanumab are recommended for the treatment of patients with confirmed Alzheimer’s disease (AD) within the mild cognitive impairment (MCI) or mild stages of dementia. Biomarker testing is required to confirm the presence of amyloid beta for prescription of DMTs. Confirmatory diagnosis at the MCI stage is important to take advantage of the narrow treatment window of currently available DMTs. We aimed to understand the current utilization and drivers/barriers for confirmatory biomarker testing and DMT treatment among MCI patients.

**Method:**

We analyzed clinical chart information and physician verbatim rationale for biomarker testing and DMT treatment decisions for 1,015 patients submitted by 151 US‐based physicians (79 Neurologists, 56 primary care physicians, 16 Geriatricians) through an online market research study. Sample composition includes 417 MCI, 416 mild dementia, and 182 moderate dementia patients based on physician assessments; patients with severe dementia were excluded from the study.

**Result:**

Patients with MCI were slightly less likely to have been tested for biomarkers (49%) than patients with mild dementia (55%); p=.09. Patients with moderate dementia were significantly less likely to have been tested compared to either group (36%); p<.05.

Similarly, DMT prescribing or planning was more common among patients with mild dementia (63%) than MCI (51%) or moderate dementia (46%); p<.05.

According to an analysis of verbatim responses from physicians, top physician barriers to proceeding with biomarker testing and/or DMT treatment in MCI include the perception that MCI is too early or mild (40%) or uncertainty about DMT risk/benefit profiles (29%).

Among MCI patients who discussed biomarker testing with their physician but were not ultimately tested, the patient made the final decision not to test in 75% of cases. The top physician‐reported patient barrier was cost/coverage concerns associated with testing and/or DMTs (55%).

**Conclusion:**

This research showed limited use of biomarker testing and DMT prescribing among MCI patients.

To address physician‐identified barriers, education for physicians and patients is needed to highlight the importance of early identification and treatment of AD. Expanded insurance coverage and support for confirmatory testing could help ease financial barriers.

